# Association between kidney intracapsular pressure and ultrasound elastography

**DOI:** 10.1186/s13054-017-1847-2

**Published:** 2017-10-19

**Authors:** Kianoush B. Kashani, Shennen A. Mao, Sami Safadi, Bruce P. Amiot, Jaime M. Glorioso, John C. Lieske, Scott L. Nyberg, Xiaoming Zhang

**Affiliations:** 1 Division of Nephrology and Hypertension, Mayo Clinic, 200 First St SW, Rochester, Minnesota 55905 USA; 2 Division of Pulmonary and Critical Care Medicine, Mayo Clinic, Rochester, Minnesota USA; 3 Division of Transplantation Surgery, Mayo Clinic, Rochester, Minnesota USA; 4 Division of Surgery Research, Mayo Clinic, Rochester, Minnesota USA; 5 Department of Radiology, Mayo Clinic, Rochester, Minnesota USA; 6 Department of Physiology and Biomedical Engineering, Mayo Clinic, Rochester, Minnesota USA

**Keywords:** Acute kidney injury, Bladder pressure, Intra-abdominal hypertension, Kidney intracapsular pressure, Swine model, Ultrasound surface wave elastography

## Abstract

**Background:**

Kidney congestion is a common pathophysiologic pathway of acute kidney injury (AKI) in sepsis and heart failure. There is no noninvasive tool to measure kidney intracapsular pressure (KIP) directly.

**Methods:**

We evaluated the correlation of KIP with kidney elasticity measured by ultrasound surface wave elastography (USWE). We directly measured transcatheter KIP in three pigs at baseline and after bolus infusion of normal saline, norepinephrine, vasopressin, dopamine, and fenoldopam; infiltration of 2-L peritoneal dialysis solution in the intra-abdominal space; and venous, arterial, and ureteral clamping. KIP was compared with USWE wave speed.

**Results:**

Only intra-abdominal installation of peritoneal dialysis fluid was associated with significant change in KIP (mean (95% CI) increase, 3.7 (3.2–4.2)] mmHg; *P* < .001). Although intraperitoneal pressure and KIP did not differ under any experimental condition, bladder pressure was consistently and significantly greater than KIP under all circumstances (mean (95% CI) bladder pressure vs. KIP, 3.8 (2.9–4.) mmHg; *P* < .001). USWE wave speed significantly correlated with KIP (adjusted coefficient of determination, 0.71; *P* < .001). Estimate (95% CI) USWE speed for KIP prediction stayed significant after adjustment for KIP hypertension (−0.8 (− 1.4 to − 0.2) m/s; *P* = .008) whereas systolic and diastolic blood pressures were not significant predictors of KIP.

**Conclusions:**

In a pilot study of the swine model, we found ultrasound surface wave elastography speed is significantly correlated with transcatheter measurement of kidney intracapsular and intra-abdominal pressures, while bladder pressure overestimated kidney intracapsular pressure.

## Background

Acute kidney injury (AKI) is a common clinical complication among patients admitted to the intensive care unit (ICU). The incidence of AKI in the hospital and the ICU has been reported as 20–45% [1, 2]. AKI is an independent risk factor for morbidity and death among patients in the ICU and hospital [3, 4]. Increased morbidity and mortality rates due to AKI correlate with the severity of renal dysfunction [5–7]. AKI is also an independent risk factor for chronic kidney disease, end-stage renal disease, and increased hospital cost [8–13]. Growing evidence shows that kidney congestion is associated with higher risk of AKI in critically ill patients who have acutely decompensated heart failure or sepsis [14]. Intra-abdominal hypertension, increased central venous pressure (CVP), and kidney mean perfusion pressure deficit have been used to indirectly predict kidney congestion and risk of AKI [15].

Compartment syndrome, as a risk factor for AKI, is characterized by increased pressure within a confined body space, with potential to compromise its microcirculation. This pathophysiologic situation most commonly occurs in fascial compartments of the limbs and the abdomen [16, 17]. Depending on its extent and duration, intracompartmental hypertension can lead to impaired blood supply, neurologic deficit, and organ or tissue damage.

Intra-abdominal pressure is the steady-state pressure concealed within the abdominal cavity [17, 18]. Intra-abdominal pressure that exceeds 12 mm Hg in critically ill patients is considered a risk factor for AKI [18–25]. Among patients with acutely decompensated heart failure, intra-abdominal pressure of 8 mm Hg has been associated with AKI [26, 27]. The pathophysiologic relationship between intra-abdominal hypertension and AKI is explained by a decrease in the filtration gradient, which is the pressure difference across the glomerular basement membrane that drives filtration [23].

To our knowledge, no noninvasive technology is available currently to measure kidney intracapsular pressure (KIP) in a clinical setting. Such a device potentially would assist clinicians to identify patients at risk for AKI from intraperitoneal hypertension and thus aid clinicians in the treatment of critically ill patients. Herein, we present results of a pilot animal study investigating the performance of a novel noninvasive tool that estimates KIP by measuring kidney elasticity. We hypothesize that KIP estimated by ultrasound surface wave elastography (USWE) would strongly correlate with directly measured KIP under different physiologic and pathologic states.

## Methods

### Experimental outline

Three healthy female pigs weighing 30–40 kg (from Manthei Hog Farm, Elk River, MN, USA) were purchased for use in this study. All animal husbandry and procedures were performed in accordance with the guidelines of the Mayo Clinic Institutional Animal Care and Use Committee (IACUC). The IACUC reviewed and approved this project (No. 130114).

All animals were sedated with short-acting injectable anesthetic (tiletamine/zolazepam 5 mg/kg) 60 minutes before the interventions. The animals were positioned supine on the operating table, and femoral arterial and central venous lines were placed under ultrasonography guidance for both monitoring and medication administration. The animals underwent endotracheal intubation and urinary indwelling catheterization. Next, they underwent bilateral flank incisions. The dissection was performed through the skin and subcutaneous tissue, avoiding injury to the peritoneum. A 22-gauge angio-catheter was positioned in each renal subcapsule under ultrasonography guidance and connected to pressure transducers. These catheters were flushed patent and without kinking. The skin was closed around the catheters with a subcuticular stitch. Bilateral KIP and bladder pressure, arterial pressure, and CVP were continuously monitored throughout each testing condition.

For evaluation of the correlation between the directly measured KIP and elastography in different physiologic and pathophysiologic states, the animals underwent individual study interventions, and pressures were documented. The interventions were chosen on the basis of their theoretical association with change in kidney hemodynamics and, therefore, in KIP. The KIP and elasticity were measured at baseline and after each individual intervention. A 20-minute period was given between each intervention to allow kidney hemodynamics to return to baseline. Table 1 summarizes the studied mechanical and pharmacologic interventions.

**Table 1:** Summary of pharmacologic and surgical interventions

Intervention	Dose	Rationale	Expected change in KIP
Baseline		Reference	↔
Perfusion with normal saline	30 mL/kg	Kidney congestion	↑
Vasopressin infusion	0.05 U/min	Kidney vasoconstriction	↓
Norepinephrine infusion	0.1 mcg/kg per min	Kidney vasoconstriction	↓
Dopamine infusion	2 mcg/kg per min	Kidney vasodilation	↑
Fenoldopam infusion	0.1 mcg/kg per min	Kidney vasodilation	↑
PD^a^ fluid installation	2 L	Intra-abdominal HTN	↑
Ureter occlusion		Obstruction	↑
Renal vein occlusion		Kidney congestion	↑
Renal artery occlusion		Kidney ischemia	↓

*Abbreviations*: *HTN* hypertension, *KIP* kidney intracapsular pressure, *PD* peritoneal dialysis

^a^PD0 is immediately after installation of PD fluid in the abdominal cavity; PD1 and PD2 are measurements recorded after 30 and 60 minutes, respectively, of PD fluid installation in the abdominal cavity

Following completion of all pharmacologic interventions, a peritoneal dialysis (PD) catheter was inserted in the low midline position. The fascia was closely approximated around the PD catheter to minimize variations in peritoneal pressure. Following PD catheter insertion, 2 L of peritoneal dialysate was instilled into the abdomen to simulate abdominal compartment syndrome. Intraperitoneal pressure measurements were repeated at installation and at 30 and 60 minutes after installation. Animals then underwent a midline laparotomy. The renal pedicles were exposed, and sequentially the ureter, renal vein, and renal artery were occluded with occlusive vascular clamps. Each occlusion step was sustained for 20 minutes. Following each step, direct renal subcapsular pressures were compared with USWE measurements. All animals were sacrificed at the conclusion of the acute study.

### Principles of USWE

**Fig. 1:**
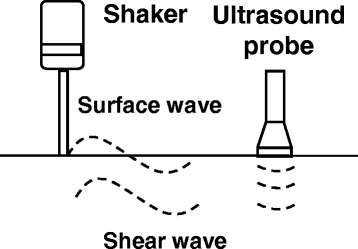
Ultrasound surface wave elastography mechanics. A vibration is generated by a shaker on the skin. The surface and shear waves can be measured with the ultrasound probe

Ultrasonography examination of the kidney can provide significant noninvasive information on the kidney and volume status of the patients who suffer from AKI [28]. Knowing the size, echogenicity, and shape of the kidney or Doppler examination of renal artery flow and measurement of the resistive index (including intraparenchymal renal resistive index variation) can provide important information on the etiology of AKI [29–31]. USWE is a novel and noninvasive technique [32, 33] for measuring the viscoelastic properties of tissue. A small hand-held probe is used to produce local harmonic vibrations of the target tissue to generate propagation of transversal or shear waves. The shear wave speed is measured with an ultrasound beam that is generated by a transducer (Fig. 1). The surface wave examines the skin while the shear wave in the tissue layer examines the deep tissue. The measurements of wave speed and wave attenuation enable calculation of viscoelastic properties. With the use of 6.5-MHz probes, the tool is able to measure shear wave speeds with the depth of 45 mm. Using lower frequency probes (i.e., 5 MHz) has the potential to assess shear wave speeds in deeper tissues. A notable advantage of USWE is its ability to measure tissue viscosity and elasticity with high accuracy and precision; both properties may be associated with disease susceptibility [34]. In addition, deep tissue layers can be analyzed to learn the contribution of various tissue types to disease manifestation. Further information on the USWE is provided in the Appendix.

Scarring and edema can change the elasticity of each individual tissue. For the organs that reside close to the surface, palpation is one of the most common physical examination tools used to evaluate the tissue elasticity. For the tissues that are located internally, use of USWE has been proposed in the recent literature [35]. Localized (i.e., neoplasm, infection, embolism, or infarction, or a combination) or diffuse (e.g., edema, inflammation, congestion, fibrosis) changes in elasticity can be detected in the kidney by elastography.

### Statistical analysis

Categorical data were summarized as number and percentage; continuous variables as mean (SD) or median (IQR), as appropriate. Measurements were divided into a binary variable based on association with intra-abdominal hypertension simulated by the installation of PD fluids in the animal abdominal cavities (i.e., measurements with peritoneal fluid classified as the KIP hypertensive group vs. those without peritoneal fluid classified as the KIP normotensive group). We used linear regression models to evaluate the correlation between wave speed and KIP after adjustments for other hemodynamic variables and the binary variable of intra-abdominal hypertension.

## Results

**Fig. 2:**
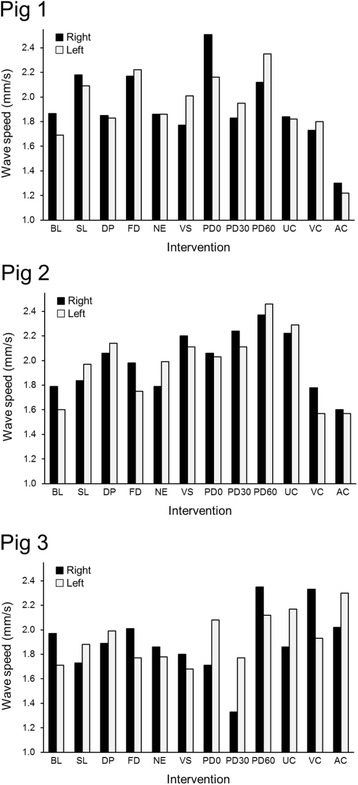
Wave speed changes of each pig, based on the intervention. PD0 is immediately after installation of peritoneal dialysis fluid in the abdominal cavity; PD30 and PD60 are measurements that are recorded after 30 and 60 minutes of peritoneal dialysis fluid installation in the abdominal cavity, respectively. AC, renal artery occlusion; BL, baseline; DP, dopamine infusion; FD, fenoldopam infusion; NE, norepinephrine infusion; PD, peritoneal dialysis; SL, saline infusion; UC, ureter occlusion; VC, renal vein occlusion; VS, vasopressin infusion

The study involved three pigs (six unique kidneys). Median and interquartile range (IQR) of monitored pressures were systolic blood pressure, 94 (88–100) mm Hg; diastolic blood pressure, 53 (50–59) mm Hg; KIP, 7 (7–10) mm Hg; bladder pressure, 7 (6–11) mm Hg; and intraperitoneal pressure, 7 (6–11) mm Hg. Median (IQR) of heart rate, temperature, and USWE wave speed were 116 (112–144) beats per minute, 36.7 °C (35.8–37 °C), and 2.01 (1.77–2.31) m/s, respectively. In order to acquire kidney images for this study, the average depth of the ultrasound (US) images was 40 mm, and the kidney surfaces were located at a depth of 25 mm. Figure 2 shows all USWE wave speed measurements in the six kidneys.

The mean (95% CI) difference between KIP and the intraperitoneal pressure was not significant (0.39 (− 0.9 to 0.2) mm Hg, *P* = .20). However, the KIP and intraperitoneal pressure differed significantly from bladder pressure (mean (95% CI) difference of bladder pressure vs. KIP was 3.8 (2.9–4.7) mm Hg, *P* < .001; mean (95% C) difference of bladder pressure vs. intraperitoneal pressure was 3.4 (2.3–4.7) mm Hg, *P* < .001).

**Fig. 3:**
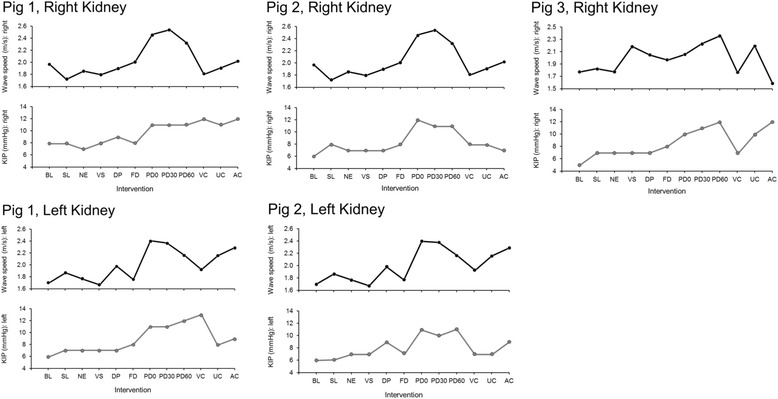
Overlay plots show correlation between wave speed and directly measured kidney intracapsular pressure (KIP). PD0 is immediately after installation of peritoneal dialysis fluid in the abdominal cavity; PD30 and PD60 are measurements that are recorded after 30 and 60 minutes of peritoneal dialysis fluid installation in the abdominal cavity, respectively. AC, renal artery occlusion; BL, baseline; DP, dopamine infusion; FD, fenoldopam infusion; NE, norepinephrine infusion; PD, peritoneal dialysis; SL, saline infusion; UC, ureter occlusion; VC, renal vein occlusion; VS, vasopressin infusion.

KIP was significantly greater during the peritoneal dialysis (PD) fluid installation phase when it was compared with the other interventions (mean (95% CI), 3.7 (3.2–4.2) mm Hg; *P* < .001). In the linear regression model, after adjustment for KIP hypertension, there was significant correlation between USWE wave speed and KIP (adjusted coefficient of determination (*r* ^*2*^) = 0.71; *P* < .001). The estimate (95% CI) of the USWE wave speed for prediction of KIP stayed significant after adjustment for KIP hypertension (− 0.8 (− 1.4 to − 0.2) m/s; *P* = .008); systolic and diastolic blood pressure were not significant predictors of KIP. The variance inflation factor of the model was 1.01, which indicates no collinearity among variables in the model. In similar models for prediction of peritoneal and bladder pressures using USWE wave speed and after adjustment for KIP hypertension, *r* ^*2*^ was 0.72 (*P* < .001) and 0.19 (*P* = .002), respectively. Figure 3 shows the changes in KIP and USWE wave speed in each kidney included in the experiment.

## Discussion

In this pilot interventional swine model experiment, we identified significant correlation between USWE and KIP (*r* ^*2*^ = 0.71). Interestingly, although we found no significant difference between KIP and intraperitoneal pressure, bladder pressure was significantly greater than both KIP and peritoneal pressure measurements. Indeed, the *r* ^*2*^ for correlation between USWE and bladder pressure was lower (*r* ^*2*^ = 0.19) than the correlation of USWE wave speed and KIP (*r* ^*2*^ = 0.71) or peritoneal pressures (*r* ^*2*^ = 0.72). Apart from the installation of PD fluid in the abdominal cavity, which was associated with a significant increase in both KIP and intra-abdominal pressure, no other intervention significantly changed KIP or abdominal pressure.

The body of evidence is growing that increased KIP is associated with higher risk of AKI. Increased KIP could be caused by congestion or intra-abdominal hypertension. Rising KIP mitigates the differences between the glomerular filtration pressure and the proximal tubular pressure—filtration gradient. This effect, in turn, decreases the glomerular filtration rate (GFR) and therefore worsens kidney function [23]. In addition, a significant increase in KIP would result in reduced renal blood flow and hence lower GFR [36]. Yet, lymphatic drainage of the kidney interstitium is jeopardized in the clinical setting of KIP hypertension [37].

In practice, the relationship between kidney congestion and AKI was described by Winton [38] in a classic study in 1935. Winton reported decreasing urine production when CVP dramatically increased in a dog model. In a more recent study, the risk of AKI rose by 2% for each 1 cm H_2_O of increased CVP [39]. Among patients with sepsis, increasing CVP was linearly associated with AKI [40]. The potential importance of increased CVP has also been suggested among patients with acute decompensated heart failure, among whom CVP greater than 8 mm Hg is associated with worsening renal function, whereas interventions to decrease CVP could increase the chances of kidney recovery [26, 27]. These examples of congestive nephropathy are commonly seen among patients who undergo aggressive fluid resuscitation or have worsening heart failure and could become worse from progressive fluid overload [41]. Currently, apart from using CVP as a surrogate for kidney congestion, no other test or tool allows clinicians to estimate KIP and identify patients at risk for congestive nephropathy.

The literature on the relationship between intra-abdominal hypertension and AKI is extensive. Sugrue et al. [19] reported on 88 patients post laparotomy with complete intra-abdominal pressure monitoring through bladder pressure. The odds ratio (95% CI) of AKI among those with intra-abdominal pressure exceeding 20 mm Hg was 12.4 (3.8–41.7). In a follow-up study of post-emergent surgical patients, the same authors [20] noted that the incidence of AKI was significantly associated with sepsis, age greater than 60 years, and intra-abdominal pressure greater than 18 mm Hg. Hering et al. [42] demonstrated that mean (SD) intra-abdominal pressure in 16 mechanically ventilated patients increased from 12 (5) mm Hg to 14 (5) mm Hg (*P* < .05) with prone positioning. In this small cohort, the authors reported a significant decrease in the renal fraction of cardiac output and renal vascular resistance index. However, they were not able to show any changes in effective renal blood flow, GFR, urine volume, and fractional excretion of sodium.

The role of intra-abdominal hypertension is well-recognized in several types of organ failure (i.e., splanchnic, respiratory, cardiovascular, and neurologic function) in addition to the kidney [23]. International conference of experts on intra-abdominal hypertension and abdominal compartment syndrome recommended monitoring intra-abdominal pressure directly (using needle puncture) or indirectly (using a balloon catheter in the bladder, stomach, rectum, inferior vena cava, or uterus) [18]. The aforementioned methods are all invasive and mostly inaccurate.

Physical examination is known to have poor sensitivity (40%) and thus is inaccurate for detection of intra-abdominal hypertension [43]. Currently, measuring intravesical (bladder) pressure is considered the gold standard for intra-abdominal pressure measurement and monitoring [43]. Almost all studies for validation of bladder pressure were done with patients who were undergoing anesthesia and paralysis for laparoscopy, which may not be a real representative of critically ill patients. Regardless, the reports of correlation between pressures measured through an intraperitoneal catheter versus measurements of bladder pressure do not have consistently high accuracy or reliability [44–46]. Importantly, in our small pilot study with a swine model, we found that bladder pressure was significantly greater than intraperitoneal pressure and KIP.

Because no noninvasive tool is available that allows clinicians to measure KIP hypertension, there is a critical need for a noninvasive device to accurately and reliably measure kidney compartmental pressure. To evaluate the potential performance of noninvasive USWE for estimation of KIP, we conducted this pilot study and noted excellent correlation between USWE and KIP or peritoneal pressure. Conversely, bladder pressure was not a reliable indicator of either KIP or peritoneal pressure. Our results may indicate that USWE has a potential role for the detection of intra-abdominal hypertension or congestive nephropathy, particularly in the clinical setting of aggressive volume resuscitation or acute decompensated heart failure.

Unlike other available tools, USWE is noninvasive, thus it could be potentially studied at the bedside to evaluate the natural history of wave speed changes during volume resuscitation in septic patients or fluid removal in patients with acute exacerbation of heart failure. Thus, USWE could potentially improve both diagnosis and fluid management among critically ill patients.

This pilot study has several limitations. The sample size for this study was small. Therefore, our findings need to be confirmed in a larger number of animals or in clinical investigations. Although installation of PD fluid increased pressures as expected, predicted changes in KIP did not occur following other interventions (Table 1). This could be due to suboptimal dosages of drugs or a short timeline within and between each intervention, or both, in this pilot study.

## Conclusion

In this swine model pilot study, we found that USWE is able to estimate KIP and intraperitoneal pressures accurately. We also demonstrated that bladder pressure does not appear to be an accurate surrogate for KIP and intra-abdominal pressure. Our results need to be confirmed in larger studies and then validated in human clinical investigations.

## Appendix

The dynamics of ultrasound surface wave elastography

Detection of wave propagation in skin and underlying tissue is guided by ultrasonography [47, 48]. This image clearly shows the skin, subcutaneous fat layer, and abdominal muscles. Tissue motion at a given location can be analyzed by cross-correlation of the ultrasound tracking beam through each specific location. For example, several points in the first muscle layer (indicated by yellow dots) were selected with image guidance; tissue motion was then measured at these points in response to local vibration (*excitation*) of the skin. The vibration is typically a continuous wave of 0.1 to 0.2 s with a frequency of 100 to 300 Hz. A high frame rate (approximately 2000 frames/s) detects tissue motion in response to the excitation. Tissue velocity is less than 1 mm/s, but the response signal is clear. Wave speed is measured by determining the change in wave phase at the other locations, relative to the first location (mean (SD) wave speed, 2.05 (0.21) m/s). Different tissue layers can be analyzed using 1 ultrasound surface wave elastography (USWE) measurement because the instrument captures all ultrasound data from the full depth. Thus, the wave speed in the skin is determined by analyzing ultrasound data directly from the skin. Wave speed is measured by the ultrasound tracking beams, and the measurement consequently is local and independent of the excitation source.

### Implementation of USWE

For the use of commercial US machines for USWE measures, there is no need to change the US hardware, although the software should be modified [49]. Ultrasound data start being collected after a function generator (FG33120A) submits digital pulses. Then, vibration synchronized signals are amplified and submitted to a handheld shaker. The US probe measures the vibration propagation speed in the skin and deeper tissues.

### Clinical applications

Clinical applications of the USWE technique have involved measurement of biomechanical properties of skin in 30 healthy volunteers and 4 patients with systemic scleroderma [34]. Both elasticity and viscosity were significantly greater in the systemic scleroderma group than the healthy group. It is, therefore, easy to discriminate fibrotic skin from healthy skin using viscoelasticity measurements. Because accurate measures of skin fibrosis disorders continue to be challenging, objective assessment of skin fibrosis using the USWE technique is a potentially valuable clinical tool that has received a quick review from physicians [50]. USWE has been used to evaluate human lung stiffness [51], myocardial tissues [51], abdominal wall tension [52], and biomechanical properties in wound healing and scar formation [53]. Noninvasive measurement of carpal tunnel pressure was reported recently [51] with analysis of changes of wave speed in the tendon. It showed that wave speed in the tendon increases linearly with carpal tunnel pressure.

## Abbreviations

AKI: Acute kidney injury
CVP: Central venous pressure
GFR: Glomerular filtration rate
IACUC: Institutional Animal Care and Use Committee
ICU: Intensive care unit
IQR: Interquartile range
KIP: Kidney intracapsular pressure
PD: Peritoneal dialysis
US: Ultrasound
USWE: Ultrasound surface wave elastography

## References

[1] Bellomo R, Kellum JA, Ronco C (2012). Acute kidney injury. Lancet.

[2] Kam Tao Li P, Burdmann EA, Mehta RL (2013). Acute kidney injury: global health alert. J Nephropathol.

[3] Ali T, Khan I, Simpson W, Prescott G, Townend J, Smith W, Macleod A (2007). Incidence and outcomes in acute kidney injury: a comprehensive population-based study. J Am Soc Nephrol.

[4] Chertow GM, Burdick E, Honour M, Bonventre JV, Bates DW (2005). Acute kidney injury, mortality, length of stay, and costs in hospitalized patients. J Am Soc Nephrol.

[5] Singbartl K, Joannidis M (2015). Short-term effects of acute kidney injury. Crit Care Clin.

[6] Ponce D, Dias DB, Nascimento GR, Silveira LV, Balbi AL (2016). Long-term outcome of severe acute kidney injury survivors followed by nephrologists in a developing country. Nephrology.

[7] Oeyen S, De Corte W, Benoit D, Annemans L, Dhondt A, Vanholder R, Decruyenaere J, Hoste E (2015). Long-term quality of life in critically ill patients with acute kidney injury treated with renal replacement therapy: a matched cohort study. Crit Care.

[8] Chawla LS, Amdur RL, Amodeo S, Kimmel PL, Palant CE (2011). The severity of acute kidney injury predicts progression to chronic kidney disease. Kidney Int.

[9] Chawla LS, Eggers PW, Star RA, Kimmel PL (2014). Acute kidney injury and chronic kidney disease as interconnected syndromes. N Engl J Med.

[10] Chawla LS, Kimmel PL (2012). Acute kidney injury and chronic kidney disease: an integrated clinical syndrome. Kidney Int.

[11] Waikar SS, Liu KD, Chertow GM (2008). Diagnosis, epidemiology and outcomes of acute kidney injury. Clin J Am Soc Nephrol.

[12] Waikar SS, Liu KD, Chertow GM (2007). The incidence and prognostic significance of acute kidney injury. Curr Opin Nephrol Hypertens.

[13] Ishani A, Xue JL, Himmelfarb J, Eggers PW, Kimmel PL, Molitoris BA, Collins AJ (2009). Acute kidney injury increases risk of ESRD among elderly. J Am Soc Nephrol.

[14] Jentzer JC, Chawla LS (2015). A clinical approach to the acute cardiorenal syndrome. Crit Care Clin.

[15] Wong BT, Chan MJ, Glassford NJ, Mårtensson J, Bion V, Chai SY, Oughton C, Tsuji IY, Candal CL, Bellomo R (2015). Mean arterial pressure and mean perfusion pressure deficit in septic acute kidney injury. J Crit Care.

[16] Mubarak SJ, Hargens AR (1983). Acute compartment syndromes. Surg Clin North Am.

[17] Tiwari A, Haq AI, Myint F, Hamilton G (2002). Acute compartment syndromes. Br J Surg.

[18] Malbrain M, Cheatham M, Kirkpatrick A, Sugrue M, Parr M, De Waele J, Balogh Z, Leppäniemi A, Olvera C, Ivatury R (2006). Results from the International Conference of Experts on Intra-abdominal Hypertension and Abdominal Compartment Syndrome. I Definitions Intensive Care Med.

[19] Sugrue M, Buist MD, Hourihan F, Deane S, Bauman A, Hillman K (1995). Prospective-study of intraabdominal hypertension and renal-function after laparotomy. Br J Surg.

[20] Sugrue M, Jones F, Deane SA, Bishop G, Bauman A, Hillman K (1999). Intra-abdominal hypertension is an independent cause of postoperative renal impairment. Arch Surg.

[21] Dalfino L, Tullo L, Donadio I, Malcangi V, Brienza N (2008). Intra-abdominal hypertension and acute renal failure in critically ill patients. Intensive Care Med.

[22] Malbrain ML, Deeren D, De Potter TJ (2005). Intra-abdominal hypertension in the critically ill: it is time to pay attention. Curr Opin Crit Care.

[23] Malbrain MNG, Chiumello D, Pelosi P, Wilmer A, Brienza N, Malcangi V, Bihari D, Innes R, Cohen J, Singer P (2004). Prevalence of intra-abdominal hypertension in critically ill patients: a multicentre epidemiological study. Intensive Care Med.

[24] Mohmand H, Goldfarb S (2011). Renal dysfunction associated with intra-abdominal hypertension and the abdominal compartment syndrome. J Am Soc Nephrol.

[25] Leblanc M, Kellum JA, Gibney RT, Lieberthal W, Tumlin J, Mehta R (2005). Risk factors for acute renal failure: inherent and modifiable risks. Curr Opin Crit Care.

[26] Mullens W, Abrahams Z, Skouri HN, Francis GS, Taylor DO, Starling RC, Paganini E, Tang WH (2008). Elevated intra-abdominal pressure in acute decompensated heart failure: a potential contributor to worsening renal function?. J Am Coll Cardiol.

[27] Mullens W, Abrahams Z, Francis GS, Taylor DO, Starling RC, Tang WH (2008). Prompt reduction in intra-abdominal pressure following large-volume mechanical fluid removal improves renal insufficiency in refractory decompensated heart failure. J Card Fail.

[28] Meola M, Nalesso F, Petrucci I, Samoni S, Ronco C (2016). Ultrasound in acute kidney disease. Contrib Nephrol.

[29] Meola M, Samoni S, Petrucci I, Ronco C (2016). Clinical scenarios in acute kidney injury: parenchymal acute kidney injury-tubulo-interstitial diseases. Contrib Nephrol.

[30] Meola M, Samoni S, Petrucci I, Ronco C (2016). Clinical scenarios in acute kidney injury-parenchymal acute kidney injury - vascular diseases. Contrib Nephrol.

[31] Samoni S, Nalesso F, Meola M, Villa G, De Cal M, De Rosa S, Petrucci I, Brendolan A, Rosner MH, Ronco C (2016). Intra-Parenchymal Renal Resistive Index Variation (IRRIV) describes renal functional reserve (RFR): pilot study in healthy volunteers. Front Physiol.

[32] Zhang X, Greenleaf JF, Pittelkow MR, Kinnick RR. System and method for non-invasively measuring tissue viscoelasticity using surface waves. In*.*; 2009, US Patent Appl Pub. No.: US2010/0010346A1. http://www.google.com/patents/US20100010346.

[33] Zhao C, Zhang X, Wang Y, Qiang B, Greenleaf JF, An KN, Amadio PC. System and method for non-invasive measurement of carpal tunnel pressure. In: US Patent. 2014. https://www.google.com/patents/US9125615?dq=US+9125615+B2&hl=en&sa=X&ved=0ahUKEwj_ktvWx-TWAhVL1oMKHet8BlIQ6AEIKDAA.

[34] Zhang X, Osborn TG, Pittelkow MR, Qiang B, Kinnick RR, Greenleaf JF (2011). Quantitative assessment of scleroderma by surface wave technique. Med Eng Phys.

[35] Emelianov SY, Lubinski MA, Weitzel WF, Wiggins RC, Skovoroda AR, O'Donnell M (1995). Elasticity imaging for early detection of renal pathology. Ultrasound Med Biol.

[36] Prowle JR, Kirwan CJ, Bellomo R (2014). Fluid management for the prevention and attenuation of acute kidney injury. Nat Rev Nephrol.

[37] Rohn DA, Stewart RH, Elk JR, Laine GA, Drake RE (1996). Renal lymphatic function following venous pressure elevation. Lymphology.

[38] Winton FR (1931). The influence of venous pressure on the isolated mammalian kidney. J Physiol.

[39] Chen KP, Cavender S, Lee J, Feng M, Mark RG, Celi LA, Mukamal KJ, Danziger J (2016). Peripheral edema, central venous pressure, and risk of AKI in critical illness. Clin J Am Soc Nephrol.

[40] Legrand M, Dupuis C, Simon C, Gayat E, Mateo J, Lukaszewicz A-C, Payen D (2013). Association between systemic hemodynamics and septic acute kidney injury in critically ill patients: a retrospective observational study. Crit Care.

[41] Marik P (2014). Iatrogenic salt water drowning and the hazards of a high central venous pressure. Ann Intensive Care.

[42] Hering R, Wrigge H, Vomwerk R, Brensing KA, Schroder S, Zirserling J, Hoeft A, Spiegel TV, Putensen C (2001). The effects of prone positioning on intraabdominal pressure and cardiovascular and renal function in patients with acute lung injury. Anesth Analg.

[43] Malbrain MLNG (2004). Different techniques to measure intra-abdominal pressure (IAP): time for a critical re-appraisal. Intensive Care Med.

[44] Fusco MA, Martin RS, Chang MC (2001). Estimation of intra-abdominal pressure by bladder pressure measurement: validity and methodology. J Trauma.

[45] Yol S, Kartal A, Tavli S, Tatkan Y (1998). Is urinary bladder pressure a sensitive indicator of intra-abdominal pressure?. Endoscopy.

[46] Johna S, Taylor E, Brown C, Zimmerman G (1999). Abdominal compartment syndrome: does intra-cystic pressure reflect actual intra-abdominal pressure? A prospective study in surgical patients. Crit Care.

[47] Zhang X, Qiang B, Hubmayr RD, Urban MW, Kinnick R, Greenleaf JF (2011). Noninvasive ultrasound image guided surface wave method for measuring the wave speed and estimating the elasticity of lungs: a feasibility study. Ultrasonics.

[48] Zhang X, Qiang B, Greenleaf J (2011). Comparison of the surface wave method and the indentation method for measuring the elasticity of gelatin phantoms of different concentrations. Ultrasonics.

[49] Zhang X, Osborn T, Kalra S (2016). A noninvasive ultrasound elastography technique for measuring surface waves on the lung. Ultrasonics.

[50] Lott JP, Girardi M (2011). Practice gaps. The hard task of measuring cutaneous fibrosis. Arch Dermatol.

[51] Zhang X, Chen S, Urban M, Kinnick R, Greenleaf J. Viscoelastic properties of myocardium tissue with surface and shear wave methods. In: Acoustics’ 08. Paris: The Journal of the Acostical Society of America. 2008;123(5):3793

[52] Qiang B, Greenleaf J, Zhang X (2010). Quantifying viscoelasticity of gelatin phantoms by measuring impulse response using compact optical sensors. IEEE Trans Ultrason Ferroelectr Freq Control.

[53] Hill D, Qiang B, Pittelkow M, Zhang X. Surface wave method analysis dynamically quantitates wound healing and scar formation. In: Society for Investigative Dermatology Annual Meeting. Raleigh: Tissue Regeneration, Stem Cells, and Wound Healing; 2012. p.107.

